# Percutaneous coronary intervention using a drug-coated balloon in a patient with haemophilia

**DOI:** 10.1093/ehjcr/ytag121

**Published:** 2026-02-17

**Authors:** Tuomas T Rissanen

**Affiliations:** Heart Center, North Karelia Central Hospital, Siun Sote Joensuu, Finland and University of Eastern Finland, FIN-80210 Joensuuu, Finland

## Case description

A young patient with type 1 diabetes, hypercholesterolaemia, hypertension, and moderately severe haemophilia A requiring factor FIII replacement therapy had exertional angina lasting for 2 months. The stress ECG testing done in a private clinic showed transient ST-elevation in the anterior leads during peak exercise, and the patient was sent to the emergency department of our hospital. Troponin-P was normal on the next day (11 ng/L). The patient was at a very high bleeding risk due to a history of several intramuscular and intra-articular bleeds leading to prosthetic replacements in his left shoulder and right ankle joints. The patient was not on any antiplatelet or antianginal medication before the stress test. LDL-C was 3.0 mmol/L and HbA1c was 69 mmol/L.

**Figure 1:**
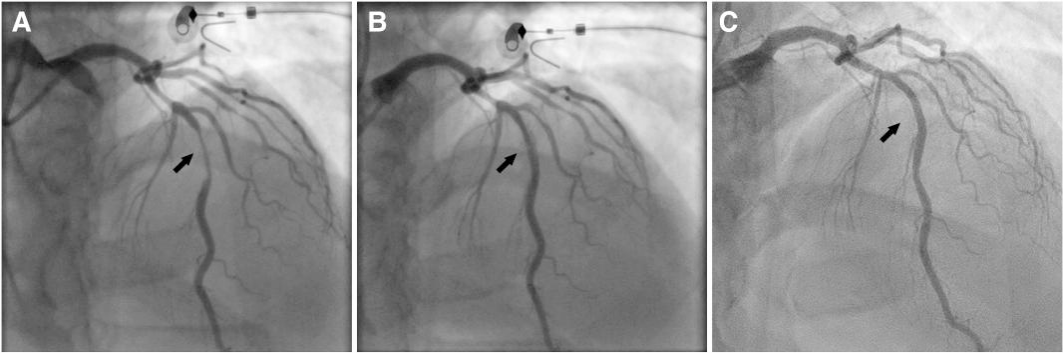
Panel *A:* Coronary angiography showed a tight stenosis (arrow) in the left anterior descending artery of a young patient with moderately severe haemophilia A. Panel *B:* A non-flow-limiting dissection was observed on post-angioplasty angiography after using a cutting and drug-coated balloon (arrow in). Panel *C:* Coronary angiography 10 years after the index procedure revealed a very good long-term therapeutic result following drug-coated balloon angioplasty with complete healing of the previously seen arterial dissection.

Coronary angiography revealed a tight stenosis in his left anterior descending artery (Panel A in *Figure 1*, supplementary material online, *Video S1*). Implantation of a drug-eluting stent combined with dual antiplatelet therapy was deferred because of the patient's extreme bleeding risk. Instead, a 3.0 × 30 mm paclitaxel-iopromide drug-coated balloon (SeQuent Please, B Braun) was used to treat the lesion to reduce the risk of restenosis compared to plain-old balloon angioplasty after predilatation with a 3.0 mm cutting balloon.^1^ After angioplasty, a non-flow limiting dissection was observed with no symptoms or ECG changes (Panel B, supplementary material online, video  *S2*). Intravascular imaging was not used. The patient was pretreated with 250 mg aspirin. After PCI, he continued using a reduced dose of 50 mg per day.^2^ No P_2_Y_12_ receptor blocker was given at any time. Aggressive secondary prevention measures were initiated, including statin therapy for cholesterol lowering, and later ezetimibe and evolocumab. The patient got a significant symptom relief after PCI.

Ten years later, the patient underwent coronary angiography for chest discomfort, which revealed a very good long-term therapeutic result following the ‘leaving nothing behind’ approach with only single-antiplatelet therapy. No significant progression of coronary artery disease was found (Panel C, supplementary material online, video  *S3*). At this time, his LDL-C was 1.0 mmol/L and HbA1c was 65 mmol/L.

Ten months after the procedure, the patient experienced an episode of haematuria but did not have bleeding or ischaemic events related to angioplasty using a drug-coated balloon with single-antiplatelet treatment.

The patient has given a written informed consent for the publication of the case report and the accompanying coronary angiography images.

## Supplementary Material

ytag121_videos_Legends

Video1

Video2

Video3

## Data Availability

No new data were generated in support of this research.

## Supplementary material

Supplementary ytag121_videos_Legends

Supplementary Video1

Supplementary Video2

Supplementary Video3
