# Supplementary material for: Percutaneous coronary intervention using a drug-coated balloon in a patient with haemophilia
Source: Eur Heart J Case Rep. 2026 Feb 17;10(3):ytag121. doi: 10.1093/ehjcr/ytag121 (PMC12985687; doi:10.1093/ehjcr/ytag121)
Supplement: ytag121_videos_Legends [file ytag121_videos_legends.docx]

**Legends for supplementary videos**

**Video 1.**

A 34-year-old male with moderately severe hemophilia A, type 1 diabetes, hypercholesterolemia had coronary angiography due to exertional angina lasting for two months. A tight stenosis was found in his left anterior descending artery.

**Video 2.**

Paclitaxel-iopromide drug-coated balloon was used to treat the lesion after predilatation with a cutting balloon. A non-flow limiting dissection in the left anterior descending artery was observed on post-angioplasty angiography with no symptoms or ECG changes. Implantation of a drug-eluting stent combined with dual antiplatelet therapy was deferred because of the patient's extreme bleeding risk.

**Video 3.**

Ten years later, the patient underwent coronary angiography for chest discomfort, which revealed a very good long-term therapeutic result with complete healing of the previously seen arterial dissection.
